# Signal-off impedimetric immunosensor for the detection of *Escherichia coli* O157:H7

**DOI:** 10.1038/srep19806

**Published:** 2016-01-22

**Authors:** Jingzhuan Wan, Junjie Ai, Yonghua Zhang, Xiaohui Geng, Qiang Gao, Zhiliang Cheng

**Affiliations:** 1 Key Laboratory of Applied Surface and Colloid Chemistry, Ministry of Education, School of Chemistry and Chemical Engineering, Shaanxi Normal University, Xi’an, 710062, China; 2 Department of Bioengineering, University of Pennsylvania, Philadelphia, PA 19104, USA

## Abstract

A signal-off impedimetric immune-biosensor based on gold nanoparticle (AuNP)-mediated electron transfer (ET) across a self-assembled monolayer (SAM) was the developed for highly sensitive detection of *Escherichia coli* O157:H7 bacteria. The biosensor was fabricated by covalently grafting an anti-*Escherichia coli* O157:H7 antibody onto SAM-modified gold electrodes. Following bacterial capture, the sensor was further modified by the gold nanoparticles (AuNPs). Due to the strong interaction between AuNPs and *Escherichia coli* O157:H7, AuNPs attached to the surface of the bacteria and acted as ET pathways across the insulating SAMs on the electrode surface, resulting in a significant reduction of the electron transfer resistance (Ret) between the [Fe(CN)_6_]^3−/4−^ redox probe in the solution and the substrate gold surface. Therefore, the attachment AuNPs to captured bacteria significantly enhanced the sensitivity for *Escherichia coli* O157:H7 bacteria detection.

Harmful pathogenic bacteria may be naturally present in food and water and can cause a variety of diseases in humans. One of the most common pathogens is *Escherichia. coli (E. coli)* O157:H7, which causes hemorrhagic diarrhea, renal failure, anemia and other serious health problems^1^. Traditional methods for *E. coli* O157:H7 detection include plate culture^2^, enzyme-linked immunosorbent assays (ELISAs)^3^, and polymerase chain reaction (PCR)^4^. However, these methods are generally tedious and time-consuming^5^. Therefore, a rapid, sensitive and specific method for the detection of *E. coli* O157:H7 is highly necessary^6^,^7^.

Impedimetric biosensors have been proven effective for the detection of pathogenic bacteria^8^. These sensors analyze the impedance of bacterial cells when they are attached to or associated with the electrodes. The capture of bacteria at the sensing surfaces can often increase interface impedance. Currently, most impedimetric biosensors are based on a “signal-on” mechanism (i.e., the signal directly correlates with the analyte concentration)^9^. However, the high background impedance from these sensors may affect their performance in several ways, including reduction in sensitivity. Several signal enhancement strategies have been studied to achieve low detection limits^10^. For example, Ruan *et al*.^11^ reported an impedimetric immunosensor for *E. coli* O157:H7 bacteria detection that used secondary antibodies labeled with horseradish peroxidase (HRP) for signal amplification. Specifically, HRP was applied to precipitate insoluble products on the electrode surface. This biosensor was able to detect *E. coli* O157:H7 bacteria with a detection limit of 6 × 10^3^ cells/mL.

Compared with enzyme-labeled assays, the use of nanomaterial tags, especially gold nanoparticles (AuNPs), can offer several benefits in terms of time, cost and simplicity^12^. For impedimetric sensors, AuNPs are often used to modify the electrode to improv substrate performance^13^. As previously reported, AuNPs can act as electron-transfer (ET) mediators across the insulating layer on the electrode surface^14^. Moreover, ET at the interface of metal NPs/insulator/metal electrode sandwich structure is more efficient than ET between a metal electrode and redox species in solution by several orders of magnitude^15^. In this study, we took advantage of the strong interaction between *E. coli* O157:H7 bacteria and AuNPs to develop a signal-off impedimetric immunosensor for the sensitive detection of *E. coli* O157:H7.

## Results and Discussion

**Figure 1:**
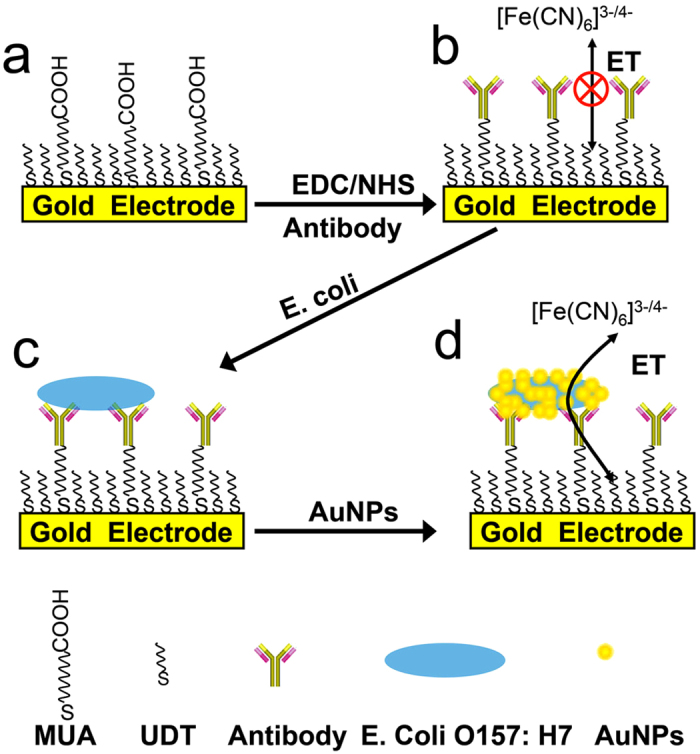
Schematic of the biosensor preparation and *E. coli* O157:H7 bacteria detection.

The development of the signal-off impedimetric immunosensor is shown in Fig. 1. Specifically, mixed self-assembled monolayers of 11-mercaptoundecanoic acid (MUA) and 1-undecanethiol (UDT) formed on the gold electrode in ethanol (EtOH). To chemically conjugate the antibody, 10 mol % COOH-terminated alkanethiol MUA was added during the self-assembled monolayer (SAM) preparation. *Anti-E. coli* O157:H7 antibody immunoglobulin G (IgG) was then conjugated onto COOH-terminated SAM-modified gold electrode based on the 1-ethyl-3-[3-dimethylaminopropyl] carbodiimide hydrochloride (EDC)/N-hydroxysulfosuccinimide (NHS) method. Because SAMs are insulators, they block ET between [Fe(CN)_6_]^3−/4−^ in an aqueous solution and the substrate gold electrode. Following antibody conjugation, bacteria were captured onto the IgG-immobilized gold electrode. To improve the detection sensitivity, electrodes covered in surface-bound bacteria were exposed to AuNPs, resulting in AuNP-coated bacteria on the electrode surface. This arrangement significantly increased ET between [Fe(CN)_6_]^3−/4−^ and the substrate gold electrode because the attached AuNPs provided an electrical pathway for electron transfer across the insulating layer on the electrode surface. The bacteria were specifically and sensitively detected by measuring the electron transfer resistance (Ret) with electrochemical impedance spectroscopy (EIS).

**Figure 2:**
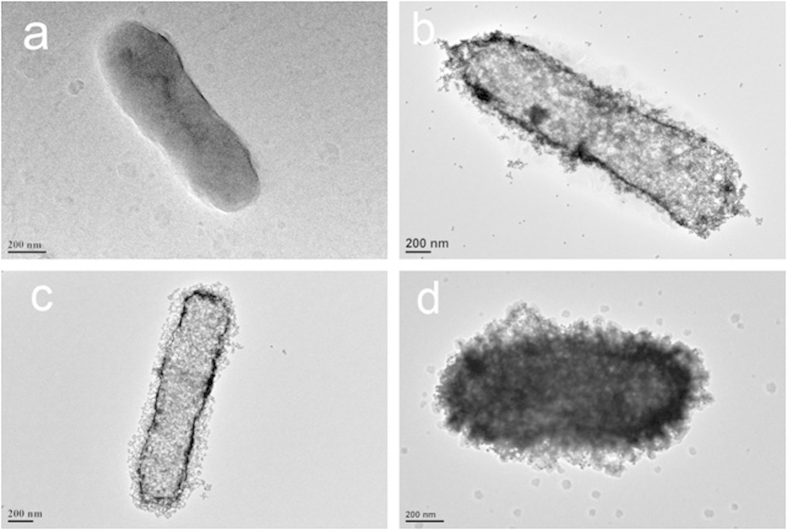
Transmission electron microscopy images of (**a**) *E. coli* O157:H7 bacteria, (**b**) AuNP-labeled *E. coli* O157:H7 bacteria without washing, (**c**) AuNP-labeled *E. coli* O157:H7 bacteria washed with PBS, (**d**) complex after the addition of HAuCl_4_ and NH_2_OH to AuNP-labeled *E. coli* O157:H7 bacteria.

Prior to coating the electrode surface-bound bacteria with AuNPs, the interaction between AuNPs and bacteria in the aqueous solution was examined. Citrate-capped gold nanoparticles with a mean diameter of approximately 13 nm were used to coat the bacteria. The successful bacterial labeling with AuNPs was confirmed by transmission electron microscopy (TEM). As shown in Fig. 2a, the unlabeled bacteria exhibited a spheroid shape (ca. 2000 nm). However, a strong interaction between AuNPs and bacteria was observed when bacteria were incubated with AuNPs. As shown in Fig. 2b, the bacteria were sufficiently coated with the AuNPs. Moreover, the AuNP-labeled bacteria retained their size and shape. The bacteria labeled with AuNPs were further washed several times with phosphate buffer solution (PBS). As shown in **sure 2c**, PBS washing did not desorb the AuNPs from the bacteria, which indicated that the AuNPs and bacteria strongly interacted. To confirm that the coated AuNPs were presented on the surface of the bacteria, i.e., not inside of the bacteria, HAuCl_4_ and NH_2_OH were added to sample of AuNP-bacteria complexes. As shown in Fig. 2d, the AuNPs were enlarged on the surface of the bacteria. Au nano-plates were also observed around the complexes^16^. These results indicated that AuNPs had mainly adsorbed to surfaces of the bacteria.

To determine the consistency and ensure that the ratio of adsorbed AuNPs to bacteria was similar for every run, TEM was used to further characterize the complexes that formed between AuNPs and different concentrations of bacteria. Under these conditions, excess AuNPs were added to bacteria. As shown in Figure S1, each complex exhibited similar amounts of adsorbed AuNPs, indicating that the bacteria concentration did not affect the ratio of AuNPs to bacteria in the presence of excess AuNPs.

**Figure 3:**
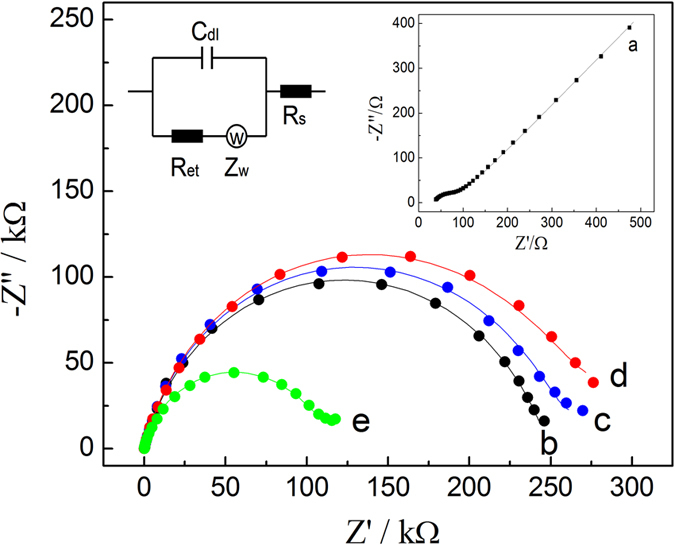
Nyquist plots of impedance spectra of (**a**) bare gold electrode, (**b**) gold electrode featuring a mixed SMA of MUA and UDT, (**c**) Antibody-conjugated electrode, (**d**) electrode coated with captured *E. coli* O157:H7 bacteria, (**e**) electrode coated with AuNP-labeled *E. coli* O157:H7.

According to the design principle of the signal-off impedimetric sensor, a highly insulating SAM on the electrode surface is critical for the sensitive detection of bacteria. Here, an insulating layer was prepared using binary SAMs consisting of MUA and UDT. EIS was used to characterize the modified processes of the impedimetric sensor, and the results are shown in Fig. 3. The EIS data were analyzed by fitting them to an equivalent electrical circuit model (inset of Fig. 3). The circuit includes the electrolyte resistance (Rs) and Warburg impedance (Zw), which results from the diffusion of ions from the bulk electrolyte to the interface, the double-layer capacitance (Cdl), and the Ret. The semicircle of the impedance spectra in the high-frequency region was attributed to the limited ET process, whereas the linear part in the lower-frequency region was attributed to transport limitations of electroactive species due to diffusion to the reaction interface, which is known as Warburg impedance. The semicircle diameter reflects the Ret, which indicates the blocking effect of the electrode surface for the [Fe(CN)_6_]^3−/4−^ redox probe. Thus, Ret was used as a sensor signal. The experimental (dots) and fitted results (lines) closely correlated.

The Nyquist plot obtained using the bare gold electrode was almost a straight line and yielded a Ret of 57 Ω, which was characteristic of a diffusion-limiting process (curve a and inset). However, a significant semicircle curve appeared in the high-frequency region for SAM-modified gold electrode (curve b). The Ret increased to 245 kΩ, indicating that electron transfer between [Fe(CN)_6_]^3−/4−^ and the electrode was strongly blocked by the SAM on the electrode. Following antibody conjugation, the Ret was 254 kΩ (curve c). Little to no change in the Ret was observed over one week when the antibody-immobilized electrodes were stored in PBS at a temperature of 4 °C. This finding indicated that the sensors were highly stable. To determine the ability of these sensors to detect bacteria, antibody-conjugated electrodes were incubated with 5000 cfu/mL *E. coli* O157:H7. The capture of *E. coli* O157:H7 to immobilized antibody only resulted in a slight increase in the Ret to 268 kΩ (curve d). This slight increase in the Ret could be due to the high background impedance from the SAM-modified electrode, which may have affected the sensitivity and detection limit for bacteria detection. To improve the performance of these sensors, the AuNPs were subsequently added to an electrode that had captured *E. coli* O157:H7. As expected, the attachment of AuNPs to the captured *E. coli* O157:H7 significantly decreased the Ret. Specifically, the Ret was changed from 268 kΩ to 106 kΩ (curve e). Therefore, the signal-off impedimetric sensor coated with AuNPs-labeled bacteria led to a 60% change in the Ret. This change was mainly due to the reduced electron transfer resistance because of *E. coli* O157:H7-adsorbed AuNPs. For comparison purposes, the signal of only the impedimetric sensor generated 6% change in the Ret when non-AuNP-labeled *E. coli* O157:H7 were captured by the antibody-conjugated electrodes. Thus, the attachment of AuNPs to *E. coli* O157:H7 led to a 10-fold signal change in bacterial detection. We also found that the physical adsorption of AuNPs to sensor surfaces without captured bacteria was very low.

To evaluate the effects of adding the AuNPs on the amount of bacteria captured on the surface, optical images of the sensor surface coated with captured bacteria were acquired before and after the addition of the AuNPs. As shown in Figure S2, the density of *E. coli O157:H7* on the sensor surface did not significantly differ between bacteria incubated with AuNPs and bacteria that had not been incubated with AuNPs, indicating that adding AuNPs did not significantly affect the amount of bacteria captured on the surface.

**Figure 4:**
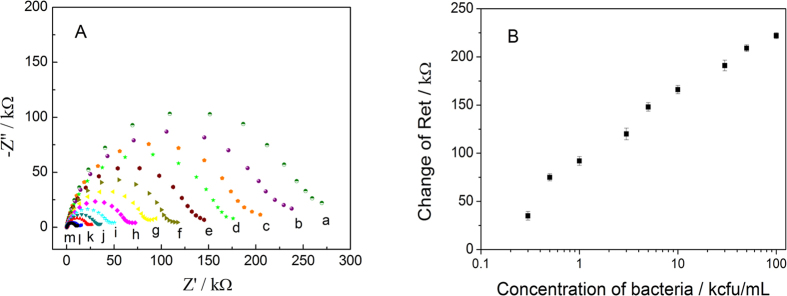
(**A**) Nyquist responses of the biosensor to different concentrations of *E. coli* O157:H7 bacteria. (a) 0, (b) 3 × 10^2^, (c) 5 × 10^2^, (d) 1 × 10^3^, (e) 3 × 10^3^, (f) 5 × 10^3^, (g) 1 × 10^4^, (h) 3 × 10^4^, (i) 5 × 10^4^, (j) 1 × 10^5^, (k) 3 × 10^5^, (l) 5 × 10^5^ and (m) 1 × 10^6^ cfu/mL. (**B**) Change in Ret as a function of *E. coli* O157:H7 concentration.

As shown in Fig. 4A, a decrease in Ret was observed when the target *E. coli O157:H7* bacteria concentration was increased from 0 to 1 × 10^6^ cfu/mL, which demonstrated that varying amounts of bacteria could be detected by measuring the Ret change. As shown in Fig. 4B, the linear dynamic range was between 300 and 1 × 10^5^ cfu/mL and characterized by linear correlation coefficient of 0.973. Each point in Fig. 4B represents the average Ret obtained with three different sensors, and error bars indicate the standard deviations of these tests. The obtained detection limit of 100 cfu/mL was based on the signal-to-noise ratio (S/N = 3).

The specificity of the biosensor for the detection of *E. coli* O157:H7 was investigated by testing non-target microorganisms, such as *E. coli DH5α, E. coli K12*, and *Staphylococcus aureus*. These organisms all produced small change s in the Ret (less than 3%), confirming that IgG-conjugated sensors specifically detected changes in the Ret due to *E. coli* O157:H7.

## Conclusion

A signal-off impedimetric immunosensor was developed for the detection of bacteria. Due to the strong interaction between AuNPs and bacteria, AuNPs easily attached to sensors that had captured bacteria and acted as ET pathways across the insulating SAMs on the electrode surface, resulting in a significant signal change. These sensors are expected to be utilized for the highly sensitive detection of other microorganisms.

## Methods

### Chemicals

11-mercaptoundecanoic acid (MUA), 1-undecanethiol (UDT), 1-ethyl-3-[3-dimethylaminopropyl] carbodiimide hydrochloride (EDC), N-hydroxysulfosuccinimide (NHS) and HAuCl_4_•3H_2_O were obtained from Sigma-Aldrich. Rabbit *anti-E. coli* O157:H7 IgG was purchased from Halin Biotech. Co. (Shanghai, China). The other chemicals and reagents were commercially available and of analytical grade. The water used was obtained from a Millipore Milli-Q purification system.

### Gold nanoparticles

Gold nanoparticles (AuNPs) with a diameter of approximately 13 nm were prepared via the citrate reduction of HAuCl_4_, as previously described^17^.

### Bacterial culture and counting

*E. coli* O157:H7 was purchased from the American Type Culture Collection. *E. coli DH5α*, *E. coli K12*, and *Staphylococcus aureus* were obtained from the China Center of Industrial Culture Collection (Beijing, China). All microbial strains were cultivated following the culturing guidelines of the manufactures and previous reports^18^. Typically, *E. coli* or *Staphylococcus aureus* cells were cultured in Luria–Bertani culture medium containing 10 g/L tryptone, 5 g/L yeast extract and 10 g/L NaCl. All strains were grown in nutrient medium in a shaking incubator (125 rpm) at 37°C. After 24 h of culturing, the bacterial cells were isolated by centrifugation (6000 rpm, 20 min) and then rinsed three times with filtered phosphate buffer solution (10 mM, pH 7.4). The number of bacterial cells was determined using the plate-counting method. The bacteria sample was directly diluted with 10 mM PBS to the desired concentrations.

### Fabrication of the biosensor

The gold electrode was sequentially polished with 1.0, 0.3, and 0.05 μm alumina slurry, followed by ultrasonic cleaning in ethanol and ultrapure water. A mixed self-assembled monolayer (SAM) was formed by immersing the electrode into the ethanol solution containing MUA and UDT at a molar ratio of 1:9 for 4 h. After washing with ethanol and buffer, the SAM-modified gold electrodes were subsequently treated with 0.05 M NHS and 0.2 M EDC solution to activate the –COOH on the electrode surface. The antibody was immobilized by dropping the antibody solution (10 μL, 1 mg/mL) onto the surface of the gold electrodes and incubating the mixture overnight at 4 °C under a humidified atmosphere. The residual activated surface was completely blocked with ethanolamine hydrochloride (1.0 mM, pH 8.5). Non-covalently bound material was removed by washing with PBS.

### Measurement of target *E. coli* O157:H7 bacteria

*E. coli* O157:H7 bacteria were captured by incubating the antibody-conjugated sensor with a bacteria sample for 1 h. After free bacteria were removed by washing with buffer, the coated electrode was then incubated in AuNP solution (8 nM) for another 1 h to attach AuNPs onto bacteria captured on the electrode surface.

### Electrochemical impedance spectroscopy (EIS) measurement

EIS was performed on a Zennium electrochemical workstation (Zahner, Germany) with a three-electrode cell. A gold electrode (2 mm in diameter) was used as the working electrode. An Ag/AgCl electrode (saturated with KCl) and a platinum wire were used as the reference and counter electrodes, respectively. All potentials in this study are presented in terms of Ag/AgCl/saturated KCl electrode potentials. The Faradaic impedance measurements were performed in the presence of a 5 mM [Fe(CN)_6_]^3/4−^ redox probe (equimolecular mixture in pH 7.0, 10 mM PBS containing 0.1 M KCl). The direct current (DC) potential was set to +0.24 V, which is equivalent to the formal potential of the [Fe(CN)_6_]^3/4−^ redox probe. The experimental data, presented as Nyquist plots, were fitted to proper equivalent circuits using the software provided by Zahner.

## Additional Information

**How to cite this article**: Wan, J. *et al*. Signal-off impedimetric immunosensor for the detection of *Escherichia coli* O157:H7. *Sci. Rep.* **6**, 19806; doi: 10.1038/srep19806 (2016).

## Supplementary Material

Supplementary Information
