# Supplementary material for: Signal-off impedimetric immunosensor for the detection of Escherichia coli O157:H7
Source: Sci Rep. 2016 Jan 22;6:19806. doi: 10.1038/srep19806 (PMC4726323; doi:10.1038/srep19806)
Supplement: Supplementary Information [file srep19806-s1.pdf]

Signal-off impedimetric immunosensor for the detection of *Escherichia coli* O157:H7

Jingzhuan Wan[1], Junjie Ai[1], Yonghua Zhang[1], Xiaohui Geng[1], Qiang Gao[1]*, Zhiliang Cheng[2]*

[1]Key Laboratory of Applied Surface and Colloid Chemistry, Ministry of Education, School of Chemistry and Chemical Engineering, Shaanxi Normal University, Xi'an, 710062, China, [2]Department of Bioengineering, University of Pennsylvania, Philadelphia, PA 19104, USA

Corresponding Author
*Qiang Gao
Tel: 86-29-81530726
Fax: 86-29-81530727
E-mail: gaoqiang@snnu.edu.cn

*Zhiliang Cheng
Tel: 215-573-0290
Fax: 215-573-2071
Email: zcheng@seas.upenn.edu

Methods

Preparation of complex AuNPs/bacteria

Bacteria medium (1 ml) containing different amounts of bacterial, 1×10^6, 1×10^5, 1×10^4 and 1×10^3 cfu, was centrifuged at 5000 rpm for 3 minutes, and then removed supernatant. Precipitation was washed with water for three times. The precipitation was then resuspended in water (1 mL). To form the complex AuNPs /bacteria, the resulting bacterial solution (50 μL) was mixted with AuNPs solution (500 μL, 8 nM) and incubated for 1 h, followed with centrifugation at 3000 rpm for 5 minutes. Precipitation was washed with phosphate buffer solution for three times and resuspended in water (1 mL). The resulting solution of the complex was used for TEM images.

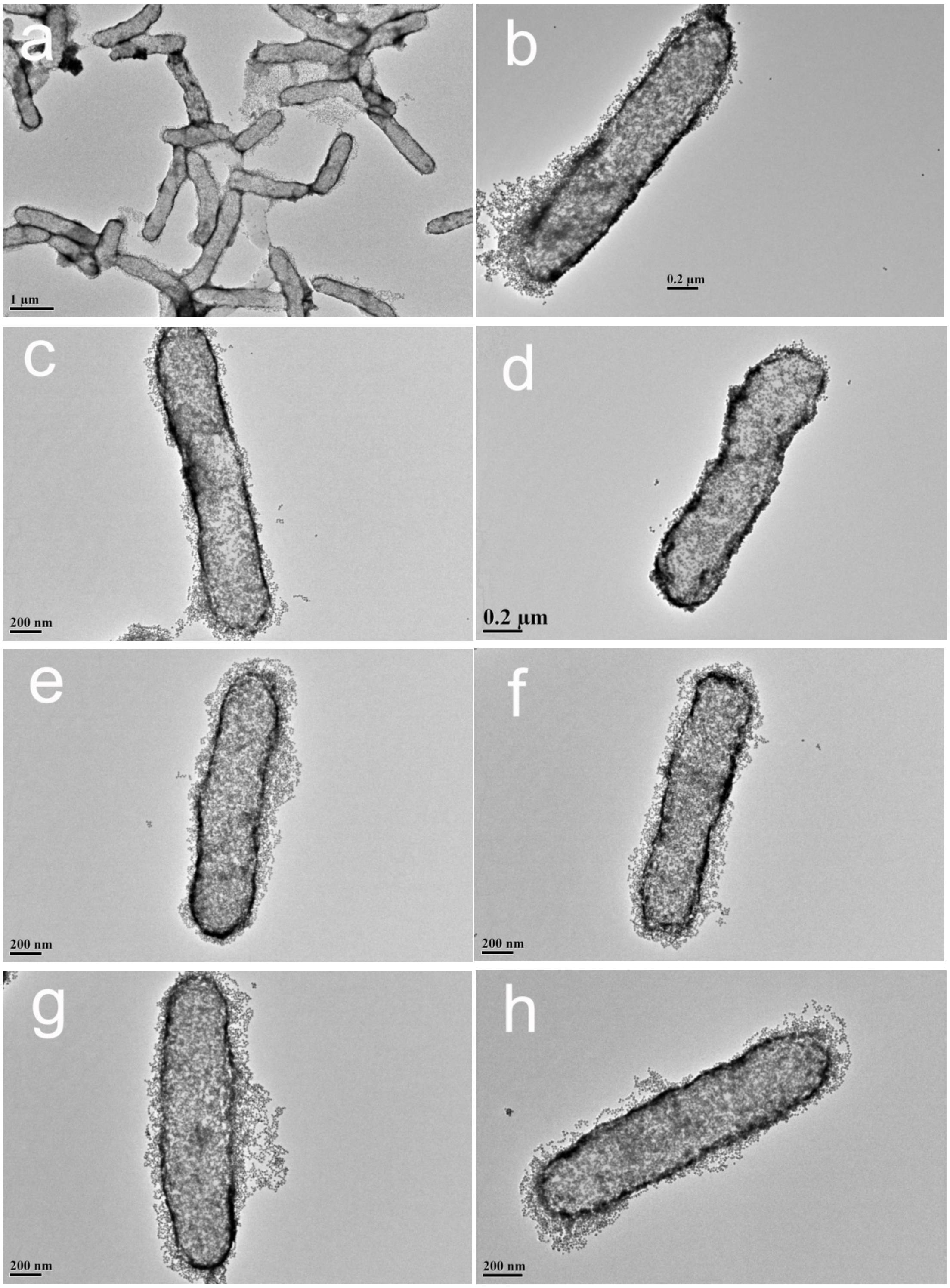

Figure S1 Transmission electron microscopy images of AuNPs/bacteria complex. The AuNPs/bacterial complex was prepared by incubation bacterail 1×10^6(a&b), 1×10^5(c&d), 1×10^4, (e&f) or 1×10^3 cfu/mL (g&h) with excess AuNPs.

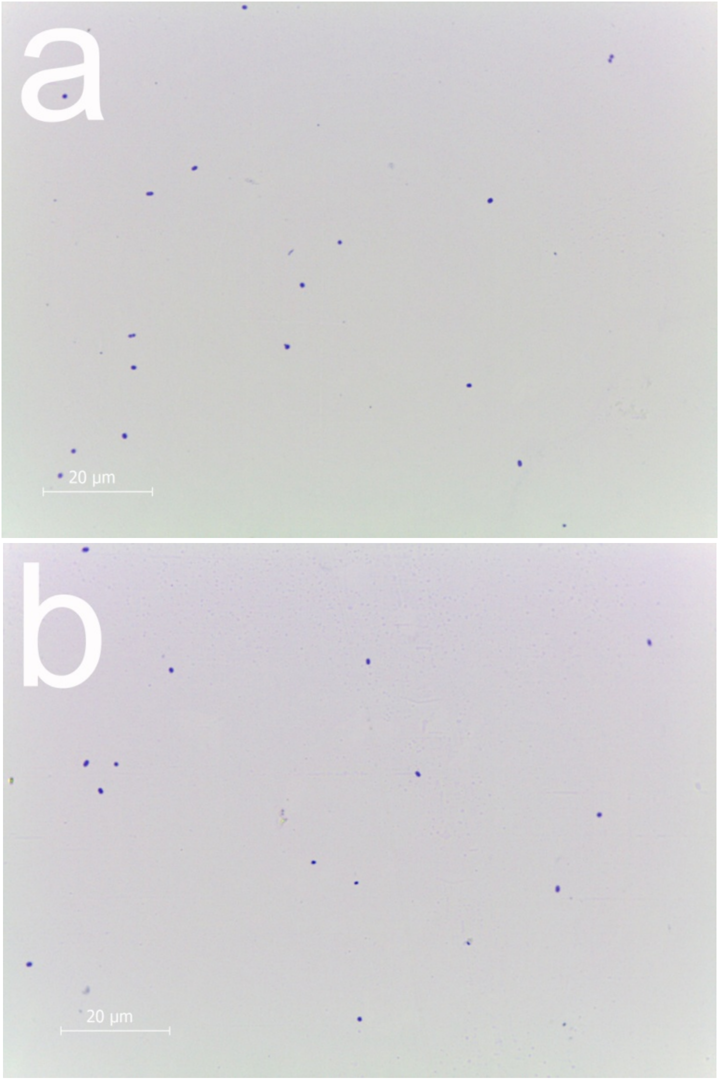

Figure S2 Optical microscope images of *E. coli O157:H7* captured on the sensor surface before (a) and after (b) immersing in AuNPs solution. 1×10^6 cfu/mL bacterial sample was used to form bacterial-captured sensor surface. The bacterial was label with crystal violet before imaging.
